# Comparative study of common over-the-counter wound care products against early and mature biofilms of antibiotic-resistant wound pathogens

**DOI:** 10.1093/jacamr/dlaf233

**Published:** 2025-12-16

**Authors:** Mohamed A N Soliman, Shivanghi Garg, Lyuboslava G Harkova, Ronan R McCarthy

**Affiliations:** Antimicrobial Innovations Centre, Division of Biosciences, Department of Life Sciences, College of Health, Medicine and Life Sciences, Brunel University London, Uxbridge UB8 3PH, UK; School of Biological Sciences, Faculty of Environmental and Life Sciences, University of Southampton, Southampton SO17 1BJ, UK; Antimicrobial Innovations Centre, Division of Biosciences, Department of Life Sciences, College of Health, Medicine and Life Sciences, Brunel University London, Uxbridge UB8 3PH, UK; Antimicrobial Innovations Centre, Division of Biosciences, Department of Life Sciences, College of Health, Medicine and Life Sciences, Brunel University London, Uxbridge UB8 3PH, UK; Antimicrobial Innovations Centre, Division of Biosciences, Department of Life Sciences, College of Health, Medicine and Life Sciences, Brunel University London, Uxbridge UB8 3PH, UK; School of Biological Sciences, Faculty of Environmental and Life Sciences, University of Southampton, Southampton SO17 1BJ, UK

## Abstract

**Background:**

The global rise of antimicrobial resistance requires innovative and affordable wound care solutions. Moreover, managing wounds infected with priority pathogens remains a challenge. Despite the widespread availability of over-the-counter (OTC) antiseptics in wound care, comparative studies on their efficacy against biofilms or multidrug-resistant pathogens are limited.

**Objectives:**

This study compares the ability of different OTC antiseptics to disrupt biofilms of multidrug-resistant clinical isolates of *Acinetobacter baumannii* and *Pseudomonas aeruginosa.*

**Methods:**

The antimicrobial activity of seven antiseptics (polyhexanide, octenidine, chloroxylenol, chlorhexidine, ethanol, cetrimide, phenol) against early-stage (3-hour) and mature (24-hour) biofilms was determined by measuring reductions in colony-forming units (cfu)/biofilm while varying treatment exposure time.

**Results:**

All OTC products significantly reduced early-stage biofilms of both pathogens below detectable limits within 5 minutes (*P* < 0.0001, *n* = 3, LOD = 100 cfu/biofilm). In mature biofilms, significant differences emerged. Polyhexanide, octenidine and cetrimide yielded modest reductions in cfu count/biofilm (0.55–0.64-log) after 5 minutes, while chloroxylenol and phenol achieved ∼2.5-log reductions; notably, chlorhexidine reduced cfu/mature biofilms below detectable limits within 5 minutes. Extended exposure (60 minutes) enhanced the efficacy of phenol and ethanol, with chloroxylenol and octenidine reducing cfu/biofilm below detectable limits.

**Conclusion:**

OTC antiseptics are effective in eliminating early-stage biofilms; however, mature biofilms require either prolonged exposure, which may increase their toxicity and delay wound healing, or the use of potent formulations. Chlorhexidine gluconate, chloroxylenol and phenol offer an optimal balance between antibiofilm potency and tissue safety, offering promise for acute and chronic wound management particularly in low-resource settings.

## Introduction

Effective frontline wound care treatments are essential in preventing and treating wound infections. Infected wounds can result in prolonged hospital stays, increased healthcare costs and increased spread of multidrug-resistant bacteria. A recent report estimated that ∼1 billion people globally suffer from acute and chronic wounds.^1,2^ The mean NHS cost for managing a non-infected surgical wound over 12 months is ∼£2,000, whereas putative infections resulting from surgical wounds can cost between £5,000 and £11,200.^3^ Another study indicated that NHS spends £8.3 billion annually for wound care, with £2.7 billion for healed wounds and £5.6 billion for unhealed ones.^4^ In the USA, wound infections influence ∼2 million individuals, compromising quality of life. They cause >200,000 deaths while wound management consumes >$18 billion annually.^5^ This highlights the critical need for timely and effective treatments to prevent wound infection or treat the infected wounds and hence enhance overall patient recovery.^6^ Advanced wound care products that are effective are often inaccessible in low-resource settings due to high costs.^7^ OTC antiseptics including octenidine dihydrochloride, polyhexanide (polyhexamethylene biguanide, PHMB), chlorhexidine digluconate, chloroxylenol, ethanol and phenols are an affordable solution which with the correct deployment have the capacity to significantly mitigate the burden of wound infections. While OTC antiseptics have been thoroughly investigated in the treatment and prevention of bacterial infections,^8–14^ there is a limited understanding of the efficacy of these antiseptics against biofilms at various stages of development.

Acute wounds are classified by their ability to heal in a timely manner to restore the structural and functional integrity of the skin and underlying tissues. Minor wounds, a subset of acute wounds, typically involve superficial injuries with minimal tissue damage.^15^ Proper management of minor wounds is essential to prevent their progression into major or chronic wounds that are more difficult to treat and significantly increase the economic burden on healthcare systems. This can be achieved through more efficacious wound care strategies to reduce the overall cost of care which can reach up to $22,130 per patient due to unhealed or infected wounds.^16^

Wound infections are often polymicrobial in nature, involving complex interactions between bacterial species that can enhance biofilm formation and resistance to treatment. A significant concern in wound management is the prevalence of commonly associated wound bacterial infections caused by ESKAPE pathogens (*Enterococcus faecium*, *Staphylococcus aureus*, *Klebsiella pneumoniae*, *Acinetobacter baumannii*, *Pseudomonas aeruginosa* and *Enterobacter* sp.).^17^  *A. baumannii* and *P. aeruginosa* rank first and second, respectively, in the WHO priority pathogen list.^18,19^ Carbapenem-resistant *A. baumannii* has been designated as one of the most critical threats among priority pathogens and a leading cause of wound infections, particularly in patients with burns and postoperative complications.^20^ A previous study showed that *A. baumannii* accounts for approximately one in five hospital-acquired infections in intensive care units, with carbapenem-resistant strains making up >13% of all intensive care unit hospital-acquired infections.^21^ Another study by Monnheimer *et al.* indicated that *A. baumannii* was isolated from wound swabs of 45 out of 301 patients in Ghana with 49% of these isolates exhibiting carbapenem resistance.^22^ Similarly, Chitandale *et al.* found that *A. baumannii* was the fourth common cause of surgical wound infections at Kamuzu Central Hospital in Malawi, with a prevalence of 12.3%.^23^ Notably, 82% of these isolates were multidrug resistant (MDR), and 14% were extremely drug resistant (XDR). In addition, a recent study by Obenhuber *et al.* found that 42.8% of patients in burns units were infected with MDR *A. baumannii*.^24^  *P. aeruginosa* is commonly responsible for chronic wound infections and the most prevalent among the Gram-negative infecting pathogens (40.2%).^25,26^ These pathogens are characterized by their ability to form biofilms that have been implicated in antimicrobial and antiseptic tolerance, complicating the treatment options and increasing the risks associated with wound healing.^27^

Open wounds lack the protective skin barrier and usually contain microorganisms from endogenous or exogenous sources. In the early stages of wound development, these microbes are typically controlled or eliminated by the host immune response.^28^ However, biofilm formation in wounds often begins when microbes adhere to the wound surface (microbial attachment stage) and then encapsulate themselves in an exopolymeric matrix of proteins, polysaccharides and bacterial DNA.^29^ This protective barrier shields the bacteria from any potential attacks by immune cells, antibodies or antimicrobials leading to localized chronic, hard-to-treat infections.^29,30^ This challenge is exacerbated by the rising rates of antimicrobial resistance worldwide with projections of >39 million deaths between 2025 and 2050 as reported by Naghavi *et al*.^31^ The study also estimates ∼1.91 million deaths directly attributed to antimicrobial resistance and 8.22 million associated deaths by 2050. However, effective management of severe infections and better access to antimicrobials could potentially revert 92 million deaths estimated between 2025 and 2050.^31,32^ Moreover, the discovery of novel antimicrobials targeting resistant Gram-negative pathogens could prevent ∼11.1 million deaths.^31,33^

Previous *in vivo* studies have demonstrated that biofilm formation in wounds can occur rapidly following bacterial infection. *S. aureus* biofilms were detected in murine wounds within 6 h of inoculation, with fibril-like and membrane-like structures forming within 1–3 h.^34^ In porcine wound models, *P. aeruginosa* developed a distinct extracellular matrix within 72 h, and *S. aureus* formed microcolonies encased in an extracellular matrix within 48 h, displaying increased antimicrobial tolerance.^35,36^ Similarly, *P. aeruginosa* established biofilms in thermally injured mouse wounds within 8 h, and *S. aureus* formed microcolonies in rabbit ear wounds within 24 h.^37,38^ In a murine model, *A. baumannii* achieved high bacterial loads (5.2 × 10⁵ cfu per 4 mm biopsy) 4 h post-infection.^39^ Collectively, these findings indicate that wound biofilms can develop within hours of bacterial colonization, underscoring the importance of early intervention. According to NHS guidance, normal saline (0.9% w/v sodium chloride) is recommended for wound cleansing in hospital settings, whereas tap water is considered suitable for wound cleaning at home. The routine use of topical antiseptics for wound cleansing is not recommended; however, their use is indicated for non-healing wounds, wounds showing signs of infection, or in cases with an increased risk of infection. Furthermore, wounds that are extensive or more than six hours old should be considered potentially colonized by bacteria, and hence the use of antiseptics may be appropriate.^40,41^

OTC antiseptics are widely available and extensively used to prevent or treat infection in non-healthcare settings, however, the use of some products is subjected to strict regulation. Under the EU Biocidal Products Regulation (EU No 528/2012) and opinion of Scientific Committee on Consumer Safety (SCCS/1581/16), PHMB is no longer approved for use in human hygiene products (PT1) due to toxicological concerns.^42–44^ Similarly, phenol and related phenolic compounds are classified as corrosive and systemically toxic under CLP/REACH (European Chemicals Agency ECHA, 2025), and in the EU it is permitted only at low concentrations (≤2.5% in consumer products and ≤1% in soaps and shampoos).^45^  ^,46^

While the antimicrobial efficacy of those OTC antiseptics is well established, their effectiveness against bacterial biofilms is not clear. Moreover, commercial products contain various excipients including surfactants, solvents and stabilizers that may influence antimicrobial activity, biofilm penetration and overall performance. Therefore, testing commercial products, rather than only the main active ingredients, provides a more clinically and practically relevant assessment.

This study aims to investigate and compare the ability of different commonly used OTC antiseptics to disrupt and eradicate both early-stage and mature biofilms associated with clinical isolates of carbapenem-resistant *A. baumannii* (AB5075) and MDR *P. aeruginosa* (PA14), two of the most prevalent wound pathogens with resistance profiles and biofilm-forming capabilities. By comparing the effectiveness against biofilm, we can evaluate their potential as practical and cost-effective solution for managing wound infections caused by antimicrobial resistant bacterial strains.

## Materials and methods

### OTC antiseptics

Elastoplast wound spray [active substance: polyhexanide (PHMB) 0.04%, other ingredients: decyl glucoside tenside 0.1% in Ringer’s solution, batch number 48389], Octenilin wound irrigation (active substance: octenidine hydrochloride, other ingredients: glycerol, ethylhexylglycerin, batch number 9460327), Dettol liquid (active substance: chloroxylenol 4.8%, other ingredients: pine oil, isopropyl alcohol, castor oil, caustic soda solution, caramel, batch number AGE687), Hibiwash skin cleanser (active substance: chlorhexidine gluconate 4%, other ingredients: poloxamer 237, isopropyl alcohol, cocamido-propylamine oxide, glycerol, macrogol 7 glycerol cocoate, gluconolactone, sodium hydroxide, batch number 5 154 257), Boots surgical spirit (ethanol 70%, batch number 44 127), Boots antiseptic liquid (cetylpyridinium chloride or cetrimide 0.025%, other ingredients: glycerol, ethanol 8.075%, sodium citrate, citric acid monohydrate, methyl salicylate, E104 quinoline yellow, batch number XLHQ7) and TCP antiseptic liquid (active substance: phenol 0.175%/halogenated phenol 0.68%, other ingredients: glycerol, concentrated phosphoric acid, E104 quinoline yellow, batch number CH434) were purchased from Boots Pharmacy, UK.

### Bacterial strains and growth conditions

Bacterial culture media were prepared and autoclaved following the supplier’s directions. Bacterial strains, multidrug-resistant clinical isolates *A. baumannii* AB5075 and *P. aeruginosa* PA14, were stored in 20% glycerol at −80°C, streaked onto agar plates and incubated for 24 h at 37°C before experiments. All experiments were carried out using LB medium and LB agar.

### Biofilm dispersal assay

From streaked agar plates, a single colony of *A. baumannii* AB5075 or *P. aeruginosa* PA14 was picked using sterile loop, inoculated into 5 mL of fresh LB medium and then incubated overnight for 18 h in a shaking incubator (180 rpm) at 37°C to prepare a bacterial suspension. The optical density OD_600_ of the overnight bacterial culture was adjusted using PBS to 0.05 (equivalent to ∼10^7^ cfu/mL). This calibration was validated for both *A. baumannii* and *P. aeruginosa* by plating serial dilutions of the adjusted cultures on LB agar and enumerating the resulting colonies.

In a 12-well plate, 1 mL of molten LB agar was added to each well and allowed to solidify. Each well was then inoculated by spotting a drop (5 µL) of diluted bacterial suspension (10^7^ cfu/mL) onto the agar surface and left to dry, see Figure S1 (available as Supplementary data at *JAC-AMR* Online). The agar was incubated at 37°C for either 3 hours (early-stage biofilms) or 24 hours (mature biofilms).^33,47–52^ The OTC treatments were used in accordance with labelled guidelines. Hibiwash and TCP antiseptic liquids were diluted with water at a 1:2 ratio while Dettol was diluted at a 1:20 ratio. Boots surgical spirit, Boots antiseptic liquid, Elastoplast spray and Octenilin wound irrigation were used undiluted as per directed use. A treatment of 1 mL was then applied into the biofilm for 5 minutes, 60 minutes and 24 hours. A negative control was used in which the biofilm was incubated for the same duration and then treated with sterile distilled water. This represents a baseline for untreated biofilm and simulates simple wound rinsing at home as recommended by NHS. After incubation, treatment was removed and the biofilm on the agar surface was resuspended by adding 1 mL of PBS and repeatedly washing the surface with the same 1 mL of PBS three times to ensure complete dispersion. The resulting suspension was serially diluted up to 10^−7^ then each dilution was spotted onto agar plates in triplicates. After 24 h of incubation, viable bacteria were enumerated to determine colony-forming units (cfu)/biofilm and log reductions were calculated relative to negative control, which reflects the bacterial load at the time of treatment. The limit of detection (LOD) for the assay was 100 cfu/biofilm.

### Statistical testing

All experiments were run in biological triplicate with three technical repeats and data were reported as mean ± standard deviation (SD). Statistical analysis using one-way analysis of variance (ANOVA) with multiple-comparison *post hoc* test was performed to evaluate the significance of the observed log reductions in the cfu count/biofilm after treatment, where the level of significance was set to *P* < 0.05.

## Results

### *OTC products were able to reduce early-stage biofilms of* A. baumannii *and* P. aeruginosa *below detectable limits*

**Figure 1.**
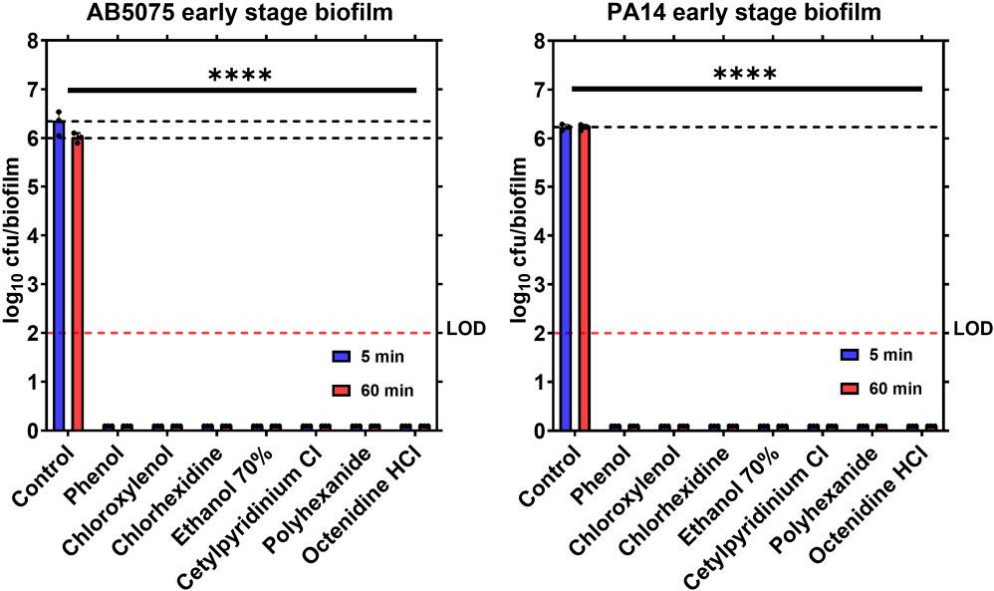
Antibiofilm activity of OTC treatments against 3-hour *A. baumannii* (AB5075), left panel, and *P. aeruginosa* (PA14), right panel, agar biofilms. The data compares the efficacy of the OTC treatments after 5 and 60 minutes of exposure to early-stage biofilm presented as viable bacterial cells remaining after treatment or number of colony-forming units (cfu) per biofilm. Results are expressed as the mean ± standard deviations (*n* = 3). The LOD for the assay was 100 cfu/biofilm. One-way ANOVA with multiple comparisons was performed to compare each treatment with the corresponding control at each time point. *P* > 0.05 was considered non-significant (ns). ****P < 0.0001.

We first evaluated the antibiofilm efficacy of seven different OTC wound care products against early-stage (3-hour) biofilms formed by carbapenem-resistant *A. baumannii* AB5075 and MDR *P. aeruginosa* PA14, See Table 1 for the OTC products’ compositions. The early-stage biofilms were validated by quantifying the viable adherent cells after 3 hours of incubating bacterial suspension in a 96-well plate at 37°C, yielding 9.78 × 10^6^ cfu/mL for *A. baumannii* AB5075 and 1.14 × 10^7^ cfu/mL for *P. aeruginosa* PA14 (see Supplementary Information S1 for Method and Figure S2). The early-stage biofilm formed on the agar surface was also assessed before treatment application, with 9.76 ×10^6^ cfu/biofilm for *A. baumannii* AB5075 and 3.56 × 10^6^ cfu/biofilm for *P. aeruginosa* PA14 were recovered. Treatment efficacy was then evaluated by recording the reduction of cfu count/biofilm after 5 minutes of applying the products, replicating a real-life wound wash scenario. A 1-mL treatment volume was selected as a representative estimate for wound washing with an antiseptic solution over 5 minutes ensuring full coverage of the agar surface and serving as a standardized volume for product comparison; however, in clinical practice, applied volumes may vary depending on wound size and depth. As a result, actual efficacy *in vivo* may be lower due to reduced exposure volume. Moreover, while prolonged exposure to OTC antiseptics is not practical in a clinical setting, a 60-minute treatment was performed to determine their maximal efficacy against early-stage biofilm. Remarkably, all OTC treatments tested reduced cfu of the early-stage biofilms for both pathogens below detectable limit within either 5 or 60 minutes (*P* < 0.0001, LOD = 100 cfu/biofilm), Figure 1. These results indicate that early-stage biofilms, lacking a well-developed exopolysaccharide matrix, are highly vulnerable to antiseptics, a finding that is consistent with previous literature for chlorhexidine and octenidine HCl.^53–56^

**Table 1.** Summary of OTC products used in the study, including their active ingredients, activity against 24-hour biofilm, reported toxicity and batch numbers

OTC products^a^	Active ingredient	Batch number	Reported toxicity	Log_10_ reduction in cfu count/biofilm after different treatment periods (*n* = 3 ± SD)					
				AB5075 24 h Biofilm^b^			PA14 24 h Biofilm^c^		
				5 min	60 min	24 h	5 min	60 min	24 h
TCP	Phenol 0.175%/halogenated phenol 0.68%	CH434	Corrosive, systemically toxic, cytotoxic to keratinocyte and fibroblast.^45,46,57^	2.55 ± 0.00	5.56 ± 0.97	ND	ND	ND	ND
Dettol	Chloroxylenol 4.8%	AGE687	Non-irritant to skin at levels up to 1% but repeated application may induce local irritation.^58^ ^,59^	2.45 ± 0.01	ND	ND	2.35 ± 0.00	5.44 ± 0.10	ND
Hibiwash Skin cleanser	Chlorhexidine gluconate 4%	5 154 257	Prolonged exposure can cause tissue irritation, cytotoxicity to host cells including human fibroblasts, myoblasts, osteoblasts.^60–62^	ND^d^	ND	ND	ND	ND	ND
Boots surgical spirit	Ethanol 70%	44 127	Tissue irritant and at > 10%: cytotoxic to human epithelial cells and fibroblasts.^63–65^	1.60 ± 0.00	5.61 ± 0.83	ND	ND	ND	ND
Boots antiseptic liquid	Cetylpyridinium chloride or cetrimide 0.025%	XLHQ7	Highest tissue toxicity across antiseptics.^66^	0.55 ± 0.00	1.82 ± 0.17	ND	1.21 ± 0.01	1.73 ± 0.10	3.44 ± 0.04
Elastoplast wound spray	Polyhexanide (PHMB) 0.04%	48 389	Cytotoxic to keratinocyte and fibroblast.^67^	0.62 ± 0.00	2.75 ± 0.21	ND	1.28 ± 0.00	2.49 ± 0.18	2.63 ± 0.04
Octenilin wound irrigation	Octenidine hydrochloride	9 460 327	Low cytotoxicity to fibroblasts and epithelial cells.^68^	0.64 ± 0.00	ND	ND	0.98 ± 0.01	5.63 ± 1.38	ND

^a^Active ingredients after dilution for use: TCP (phenol 0.058%/halogenated phenol 0.227%), Hibiwash (chlorhexidine gluconate 1.333%), Dettol (chloroxylenol 0.22%). Other products used as received without dilution.

^b^The mature biofilm of AB5075 on agar surface before applying treatment had ∼1.18 ×10^9^ cfu/biofilm.

^c^The mature biofilm of PA14 on agar surface before applying treatment had ∼2.58 × 10^9^ cfu/biofilm.

^d^ND denotes no detectable cfu where the LOD for the assay was 100 cfu/biofilm.

### *OTC products activity against mature biofilms of* A. baumannii *and* P. aeruginosa

The activity of the OTC products was further examined on more established (24-hour) biofilms to mimic a later stage of wound infection. These biofilms were confirmed by enumerating the viable adherent cells after 24 hours of incubating bacterial culture in a 96-well plate at 37°C, with 1.4 × 10^7^ cfu/mL and 1.64 × 10^7^ cfu/mL recovered from *A. baumannii* AB5075 and *P. aeruginosa* PA14, respectively (see Supplementary Information S1 for Methods and Figure S2). Further validation was performed by visualizing exopolysaccharide staining on Congo Red Agar plates (see Supplementary Information S1 for Methods and Figure S3). The mature biofilm formed on the agar surface was also quantified before applying the treatment, with 1.18 ×10^9^ cfu/biofilm for *A. baumannii* AB5075 and 2.58 × 10^9^ cfu/biofilm for *P. aeruginosa* PA14 recorded.

**Figure 2.**
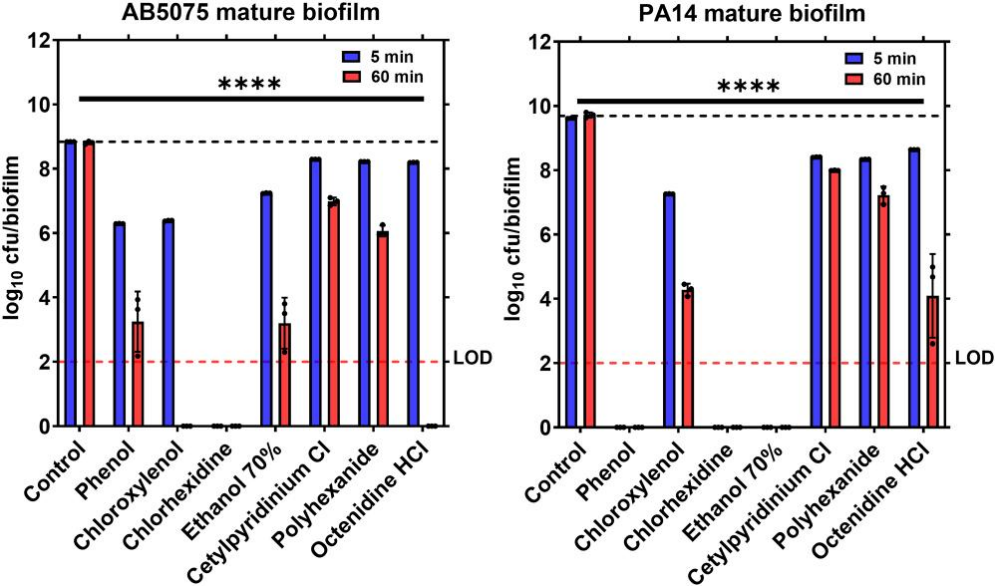
Antibiofilm activity of OTC treatments against 24-hour *A. baumannii* (AB5075), left panel, and *P. aeruginosa* (PA14), right panel, agar biofilms after 5 and 60 minutes of treatment exposure. The data compares the efficacy of the OTC treatments after 5 and 60 minutes of exposure to mature biofilm presented as viable bacterial cells remaining after treatment (number of colony-forming units (cfu) per biofilm). Results are expressed as the mean ± standard deviations (*n* = 3). The LOD for the assay was 100 cfu/biofilm. One-way ANOVA with multiple comparisons was performed to compare each treatment with the corresponding control at each time point. *P* > 0.05 was considered non-significant (ns). ****P < 0.0001.

**Figure 3.**
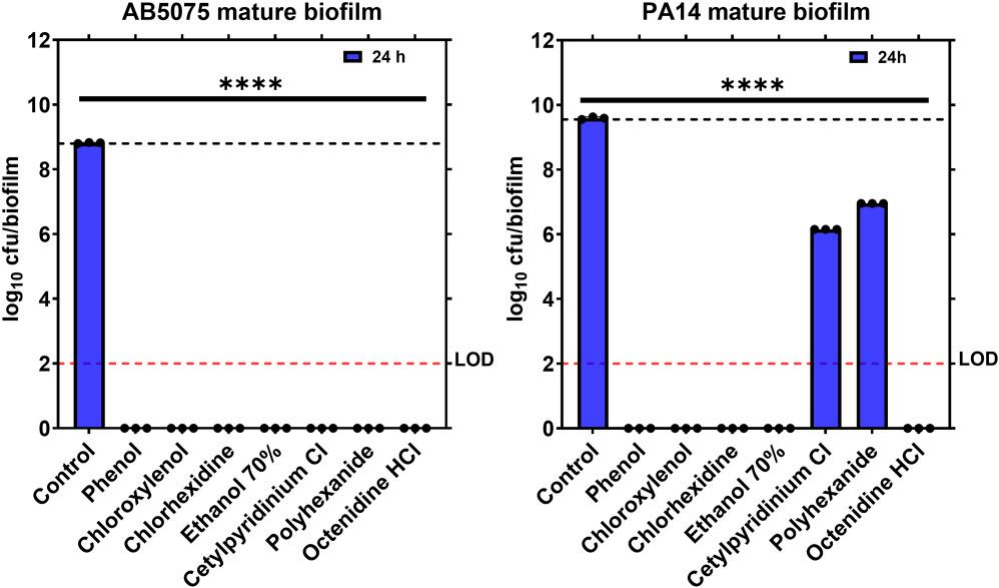
Antibiofilm activity of OTC treatments against 24-hour *A. baumannii* (AB5075), left panel, and *P. aeruginosa* (PA14), right panel, agar biofilms after 24-hour of treatment exposure. The data compares the efficacy of the OTC treatments after 24-hour of exposure to mature biofilm presented as viable bacterial cells remaining after treatment (number of colony-forming units (cfu) per biofilm). Results are expressed as the mean ± standard deviations (*n* = 3). The LOD for the assay was 100 cfu/biofilm. One-way ANOVA with multiple comparisons was performed to compare each treatment with the corresponding control at each time point. *P* > 0.05 was considered non-significant (ns). ****P < 0.0001.

At this later stage of development, significant differences in disinfectant efficacy were detected (Table 1 and Figures 2 and 3). For *A. baumannii* AB5075, a 5-minute treatment resulted in least reductions in cfu count/biofilm by ∼0.55 ± 0.00–0.64 ± 0.00 log_10_ for polyhexanide, octenidine and cetylpyridinium chloride, whereas ethanol 70% achieved an ∼1.6 ± 0.00 log_10_ reduction. More substantial reductions (∼2.45 ± 0.01 and ∼2.55 ± 0.00 log_10_ in number of cfu/biofilm) were observed with chloroxylenol and phenol, respectively. Strikingly, chlorhexidine reduced cfu/mature biofilm below detectable limit within 5 minutes (Table 1 and Figure 2, left panel). As mentioned earlier, prolonged treatments with OTC products for 60 minutes and 24 hours were conducted to evaluate their maximal efficacy against mature biofilms although these prolonged treatments are not feasible in actual wound care due to cell toxicity. For *A. baumannii* mature biofilms, a 60-minute exposure with phenol and ethanol achieved significant reductions in cfu count/biofilm exceeding 5 log_10_, while both chloroxylenol and octenidine reduced the cfu/biofilm below detectable limit. However, cetylpyridinium chloride and polyhexanide continued to show lower efficacy than other OTC products tested with ∼1.82 ± 0.17 log_10_ and ∼2.75 ± 0.21 log_10_ reductions in cfu count/biofilm, respectively (Table 1 and Figure 2, left panel). When the treatment duration was extended to 24 hours, all OTC products reduced the cfu/mature *A. baumannii* biofilm below the detectable limit (Table 1 and Figure 3, left panel).

A similar yet distinct pattern was observed with *P. aeruginosa* mature biofilms when treated with OTC wound care. Within 5 minutes of treatment, chlorhexidine, phenol and ethanol reduced the cfu/mature PA14 biofilm below detectable limit, whereas chloroxylenol and octenidine were less effective causing ∼2.35 ± 0.00 log and ∼0.98 ± 0.01 log_10_ reductions in cfu count/biofilm, respectively (Table 1 and Figure 2, right panel). Enhanced antibiofilm activity was observed after 60 minutes with chloroxylenol and octenidine, achieving reductions of ∼5.44 ± 0.10 log_10_ and ∼5.63 ± 1.38 log_10_, respectively, culminating in reducing cfu/biofilm below detectable limit within 24 hours. In contrast, cetylpyridinium chloride and polyhexanide remained the least effective against PA14 biofilms at both 60-minute (Figure 2, right panel) and 24-hour (Figure 3, right panel) treatment durations, with ∼3.44 ± 0.04 log_10_ and ∼2.63 ± 0.04 log_10_ reductions in number of cfu, respectively, after 24 hours of treatment (Table 1). These findings highlight the superior effectiveness of chlorhexidine, reducing the cfu/mature biofilms formed by both pathogens below detectable limit in 5 minutes while phenol, chloroxylenol, ethanol and octenidine needed longer exposure time (60 minutes) to show similar efficacy. It also highlights that biofilms formed by different high-priority pathogens display different levels of disinfectant tolerance.

## Discussion

While previous studies have widely investigated the antimicrobial efficacy of different OTC products, there are limited comparative data on their effectiveness against biofilms. This study aimed to explore and compare the therapeutic potential of commonly used OTC wound care products for treating acute and chronic wound infections of resistant bacterial strains of *A. baumannii* and *P. aeruginosa*. Our findings showed that all tested OTC antiseptics are highly effective against early-stage biofilms of both *A. baumannii* and *P. aeruginosa* within 5 minutes. This underscores the clinical importance of prompt wound cleansing immediately after injury to prevent initial colonization and biofilm establishment. This is particularly important when dealing with antibiotic-resistant pathogens such as *A. baumannii* and *P. aeruginosa*, where preventing biofilm formation can help mitigate the risk of persistent, hard-to-treat infections.

However, when addressing mature biofilms (>24 hours), the results diverged significantly depending on the antiseptic composition and exposure time. Among the products evaluated, chlorhexidine gluconate emerged as exceptionally potent, reducing cfu/mature biofilms of both pathogens below detectable limits within a brief 5-minute exposure. Chlorhexidine is well-documented for its broad-spectrum bactericidal effects through penetration of outer and inner cell membranes.^69,70^ Nonetheless, the cytotoxicity of chlorhexidine is a significant concern. Studies have demonstrated that prolonged exposure to chlorhexidine can lead to tissue irritation, delayed wound healing and potential cytotoxic effects on host cells (Table 1).^60–62^ Consequently, while chlorhexidine gluconate may be advantageous for short-term immediate decontamination, its use on open wounds over extended periods should be carefully managed.

Additional comparisons among the tested antiseptics revealed notable differences. Products such as chloroxylenol, phenol and octenidine hydrochloride outperformed cetylpyridinium chloride and polyhexanide in treating mature biofilms. Chloroxylenol demonstrated strong antibiofilm activity with extended exposure times (ranging from 60 minutes to 24 hours), although repeated application may induce local irritation (Table 1).^58^ The phenolic antiseptic also showed significant efficacy against biofilm, making it a potential candidate for managing both acute and chronic wounds if its toxicity remains moderate (Table 1). Octenidine hydrochloride, not only matched the efficacy of chloroxylenol but also can maintain an optimal safety profile with its reported low cytotoxicity, even during prolonged use (Table 1).^71^ By contrast, while 70% ethanol effectively reduced mature biofilm burdens and demonstrated strong *in vitro* activity, its use on open wounds is not recommended. This is due to its propensity to cause tissue irritation, which can impair wound healing and damage regenerating tissue (Table 1).^63^ Moreover, ethanol has been found to increase the virulence of *A. baumannii* by serving as a carbon source, which enhances the metabolic capacity of *A. baumannii*, induces overexpression of stress response factors and upregulates efflux pumps.^72–74^ Ethanol can also induce *A. baumannii* biofilm formation, further contributing to the resistance of *A. baumannii.*^72–74^ Cetylpyridinium chloride and polyhexanide exhibited lower antibiofilm activity and thus should be less suitable for treating infections involving mature biofilms of resistant pathogens.

These observations are consistent with previously reported studies. Chlorhexidine showed the highest activity on immature biofilms of *A. baumannii* when compared with octenidine and polyhexanide in a study reported by Denysko *et al.*^20^ Günther *et al.* also compared the capability of octenidine, chlorhexidine, chloroxylenol and polyhexanide to inhibit the metabolism of biofilm-forming clinical isolates.^75^ They recorded that octenidine and chlorhexidine had the highest activity against *A. baumannii* and *P. aeruginosa* biofilms while chloroxylenol and polyhexanide were less effective. It is worth noting that Günther *et al.* assessed the conversion of resazurin to resorufin to reflect surviving bacteria in 18 hour biofilms. Krasowski *et al.* also reported the ability of octenidine to completely remove 24 hour biofilms of *P. aeruginosa* PRT1-9.^76^ By contrast, Bonez *et al.* reported that chlorhexidine was less effective against bacterial biofilms of *P. aeruginosa* ATCC 27 853 and clinical isolates of *A. baumannii*, however, the highest concentration used in that study was 0.93%, which is lower than the one used in our study (1.33%) after dilutions recommended by the manufacturer.^77^ In another study, polyhexanide showed lower MIC and MBC (2 mg/mL) than chlorhexidine (32 mg/L) against *P. aeruginosa* ATCC 15 442; however, this was tested on a planktonic population.^78^

The findings of our study hold particular significance for wound management in low- and middle-income countries (LMICs), where major wounds and surgical site infections pose a growing challenge due to understaffing, insufficient staff training, inadequate infection control and limited healthcare resources.^79–81^ In these countries, infections rank as the second leading cause of death within the first month after birth and surgical site infections remain a leading cause of hospital-acquired infections, with rates reported between 12% and 39%.^82^ Interestingly, all OTC antiseptics investigated in this study are commercially available in LMICs, which could provide a practical and affordable solution for improved wound care and infection control.^82–84^

In summary, although all OTC products effectively targeted early biofilms, their performance against mature biofilms and differing toxicity profiles must be considered in clinical decision making. While chlorhexidine gluconate offers rapid and potent antibiofilm action, its potential cytotoxicity necessitates cautious short-term use. By contrast, octenidine, along with chloroxylenol and phenol, presents balanced alternatives suitable for both acute and chronic wound management when applied cautiously. However, it is important to note that repeated or prolonged use of octenidine or the exposure to low levels of chlorhexidine has been associated with bacterial adaptation and reduced susceptibility. Moreover, octenidine can increase bacterial tolerance to chlorhexidine, underscoring the need for careful monitoring and rational use in clinical settings.^85–88^

### Conclusion

This study demonstrates that OTC antiseptic products are highly effective against early-stage biofilms of carbapenem-resistant *A. baumannii* (AB5075) and MDR *P. aeruginosa* (PA14), underscoring the importance of prompt wound cleansing. For mature biofilms, extended exposure times or more potent formulations are required. Although chlorhexidine gluconate offers exceptional antibiofilm activity, its chlorhexidine content raises concerns regarding cytotoxicity with prolonged use on open wounds. Among the products tested, octenidine appears to offer the best balance between efficacy and safety, with chloroxylenol and phenol also being effective alternatives. Conversely, products such as cetylpyridinium chloride, polyhexanide and ethanol should be used with caution due to their lower efficacy and potential for tissue damage. Importantly, the accessibility to these OTC products in LMICs, while maintaining appropriate use, underlines their potential in enhancing wound care. This study focused only on the bacterial strains *A. baumannii* and *P. aeruginosa*, so future research should also consider the rest of the ESKAPE pathogens and polymicrobial biofilms, where Gram-positive bacteria and mixed-species communities may exhibit distinct responses to antiseptic treatments. Repeated applications and the validation of these results in more complex physiologically relevant models such as *ex vivo* wound skin and *in vivo* animal models could be also performed.

## Supplementary Material

dlaf233_Supplementary_Data

## Data Availability

All data generated or analysed during this study are included in this published article and its Supplementary Information.

## Supplementary data

Figures S1 to S3 and Supplementary Information S1 are available as Supplementary data at *JAC-AMR* Online.
