# Supplementary material for: Comparative study of common over-the-counter wound care products against early and mature biofilms of antibiotic-resistant wound pathogens
Source: JAC Antimicrob Resist. 2025 Dec 16;7(6):dlaf233. doi: 10.1093/jacamr/dlaf233 (PMC12706473; doi:10.1093/jacamr/dlaf233)
Supplement: dlaf233_Supplementary_Data [file dlaf233_supplementary_data.zip › Supplementary information 1_MS.docx]

**Supplementary Methods**

**Biofilm development assay**

The progression of biofilm formation was monitored over time. Overnight bacterial cultures were diluted in LB broth to an initial OD_600_ of 0.05 and added into 96 well plates (100 μL per well). The plates were incubated at 37 °C for 3, 6, or 24 h. At the corresponding time point, the supernatant containing planktonic cells was carefully removed, and the wells were washed three times, each time with 100 μL of PBS to eliminate non-adherent bacteria. The biofilm was then resuspended in 100 μL of fresh PBS, serially diluted, and plated on agar to determine viable cell counts (cfu/mL).

**Congo red exopolysaccharide assay**

Mature biofilm formation was assessed via exopolysaccharide production. Overnight bacterial cultures of *A. baumannii* AB5075 and *P. aeruginosa* PA14 were adjusted to OD_600_=1 and spot plated in triplicates onto agar plates of 1% tryptone, 1% agar with 40 μg/mL Congo red dye. The plates were left to air dry before incubation at 37 °C for 24 h. Images of the plates were taken after incubation, and the assay was repeated three independent times.

**Supplementary Figures**


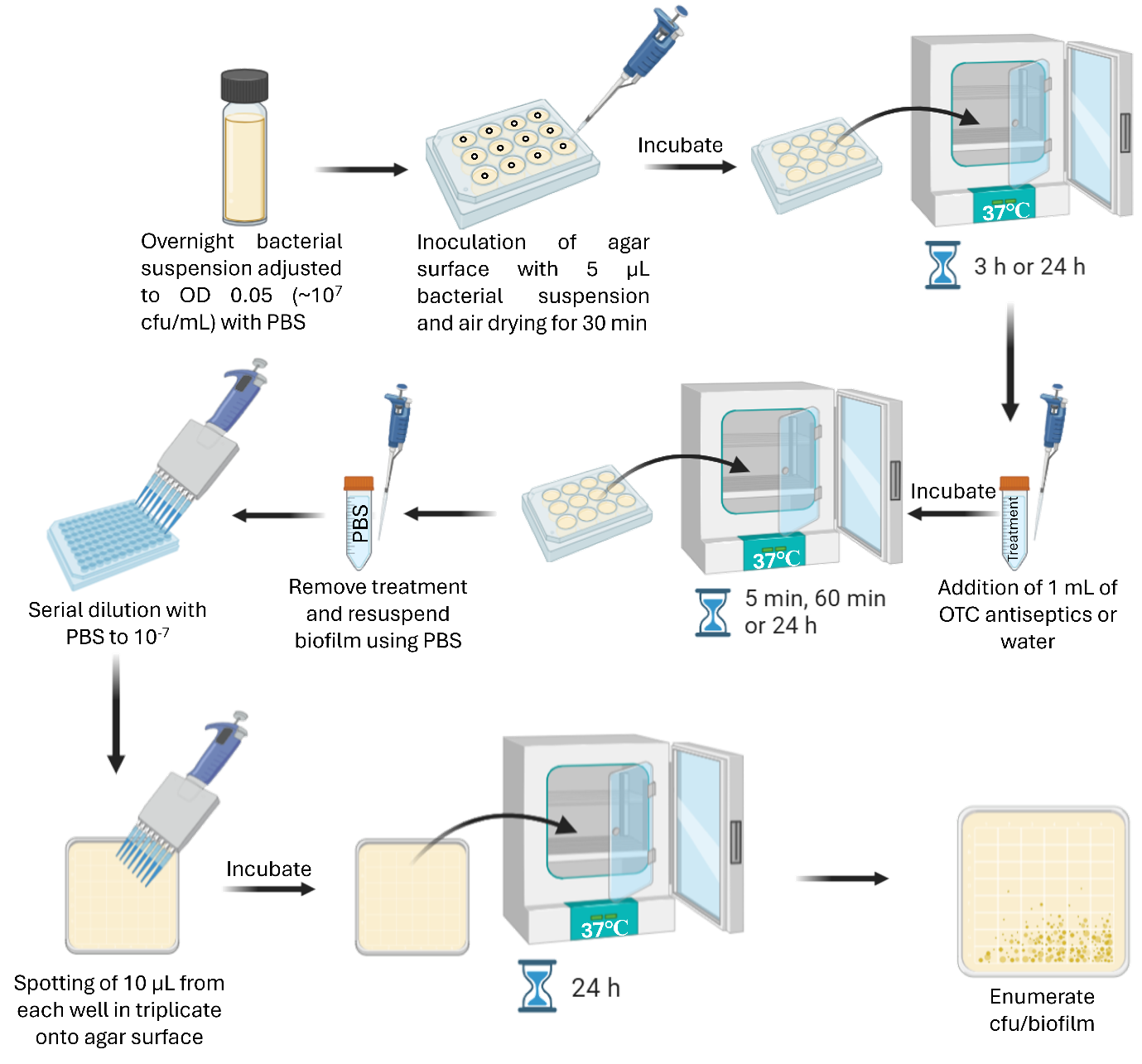


***Supplementary Figure 1. Schematic of biofilm dispersal assay.*** Agar biofilm development, treatment application, resuspension of remained biofilm and enumeration of cfu/biofilm.


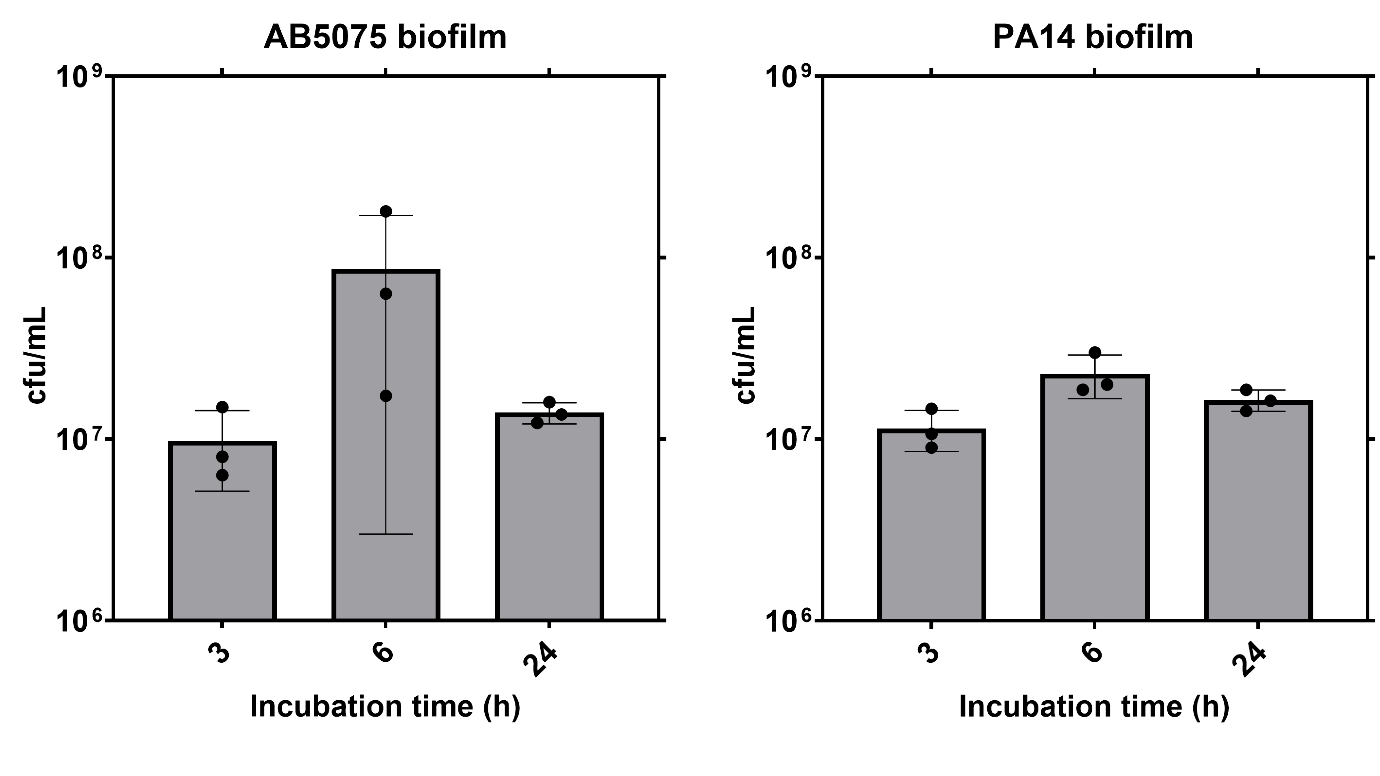


***Supplementary Figure 2. Stages of biofilm development of A. baumannii (AB5075), left panel, and P. aeruginosa (PA14), right panel, at 37℃ under static conditions in a 96-well plate.*** The data compares biofilm formed after 3h, 6h, 24h presented as viable bacterial cells remaining after treatment or number of colony-forming units (cfu)/mL. Results are expressed as the mean +/- standard deviations (n=3).

*A. baumannii* AB5075 *P. aeruginosa* PA14


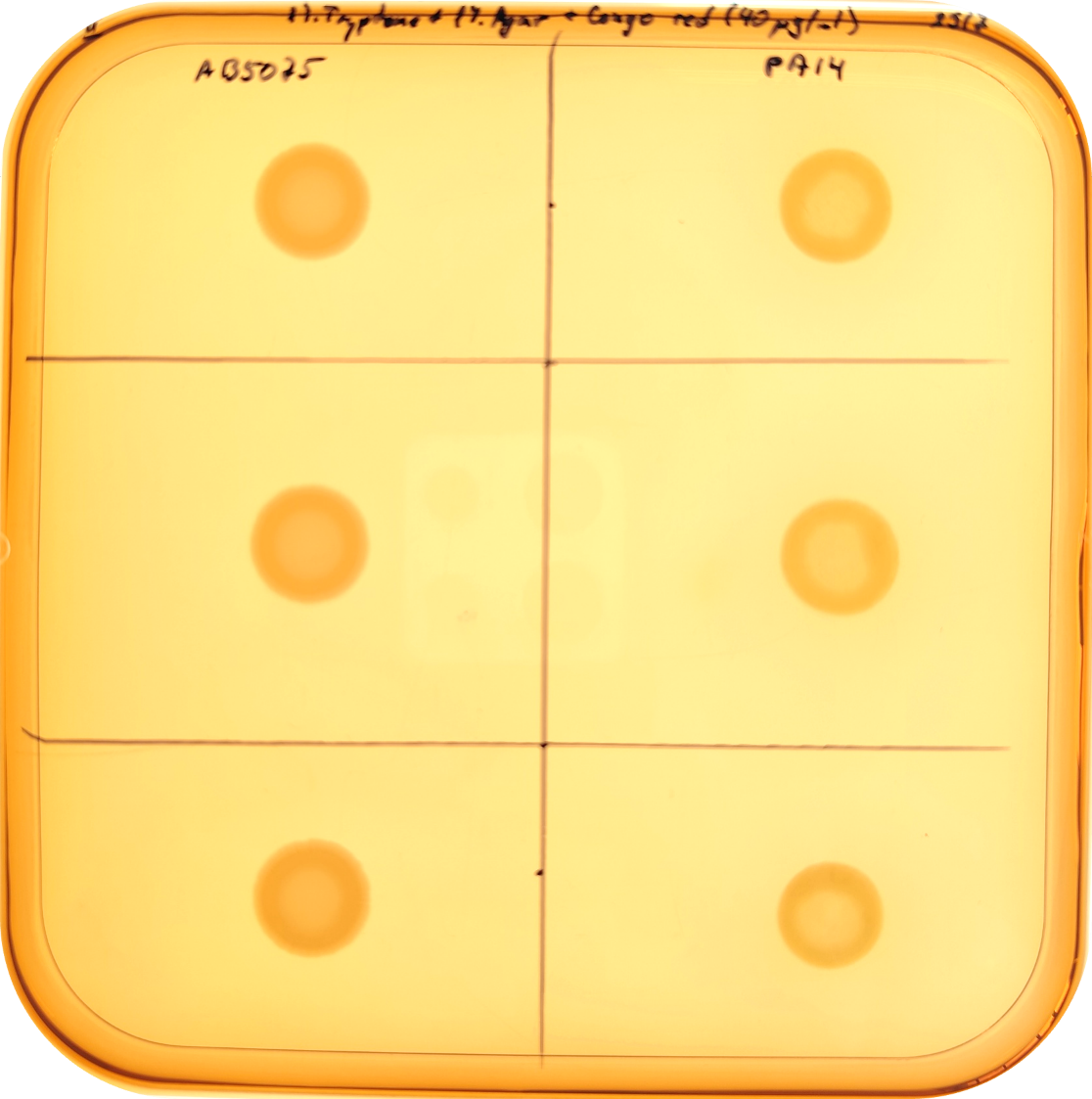


***Supplementary Figure 3. Congo Red Exopolysaccharide Assay*.** EPS assay on 1% tryptone and 1% agar with 40 µg/ml Congo red. Saturated bacterial cultures of A. baumannii AB5075 and P. aeruginosa PA14 were adjusted to OD_600_=1 and spot plated in triplicates and air-dried. Images of the plates were taken after 24 h incubation at 37 °C. Congo red staining (red, stronger at colony edges) indicates EPS production. The assay was repeated three independent times.
